# Rhapontigenin alleviates cellular senescence and physiological aging by upregulating sirt1 and promoting autophagy

**DOI:** 10.1186/s13020-025-01319-3

**Published:** 2026-01-09

**Authors:** Shuang Liu, Wendi Chen, Guoqiang Xu, Xin Liu, Yuxuan Shi, Guolong Wang, Yunna Ning, Yongzhi Cao, Ming Li, Yueran Zhao

**Affiliations:** 1 State Key Laboratory of Reproductive Medicine and Offspring Health, Center for Reproductive Medicine, Institute of Women, Children and Reproductive Health, Shandong University, Jinan, China; 2 National Research Center for Assisted Reproductive Technology and Reproductive Genetics, Shandong University, Jinan, Shandong China; 3 Key Laboratory of Reproductive Endocrinology (Shandong University), Ministry of Education, Jinan, Shandong China; 4 Department of Clinical Laboratory, Shandong Provincial Hospital Affiliated to Shandong First Medical University, Jinan, China

**Keywords:** Rhapontigenin, Cellular senescence, Antiaging, Autophagy, Sirt1

## Abstract

**Background:**

Aging is characterized by cellular senescence, inflammation, and physiological decline. Currently available antiaging therapies often have limitations due to their toxicity and off-target effects. However, natural compounds derived from Chinese herbal medicine, such as Rhapontigenin (Rhap), have shown potential as safer antiaging agents.

**Purpose:**

This study aimed to evaluate the potential of Rhap to be used as an antiaging agent by investigating its effects on cellular senescence, physical function, immune modulation, and autophagy in both in vitro and in vivo aging models.

**Methods:**

NIH3T3 and IMR90 cells were subjected to oxidative or genotoxic stress to induce senescence and then treated with Rhap. Senescence markers, cell viability, and autophagy-related protein levels were assessed. Aged mice were treated with Rhap for 8 weeks, and physical performance, immune modulation, and organ health were evaluated. Mechanistic studies were conducted to determine the role of Sirt1 in mediating the effects of Rhap.

**Results:**

Rhap treatment significantly reduced cellular senescence marker (p16 and p21) levels and senescence-associated β-galactosidase (SA-β-Gal) activity in stressed cells. In aged mice, Rhap improved physical performance, such as grip strength and motor coordination, and reduced depressive-like behaviors. Rhap also decreased liver senescence, lipid accumulation, and fibrosis and increased immune function by reducing proinflammatory cytokine production and enhancing T-cell homeostasis. Mechanistically, Rhap upregulated Sirt1 and promoted autophagy, both of which contributed to its antiaging effects. Sirt1 knockdown attenuated the effects of Rhap on autophagy and senescence, indicating the importance of Sirt1 in mediating these beneficial effects.

**Conclusion:**

Rhap is a promising candidate for mitigating age-related cellular and physiological decline by reducing cellular senescence, promoting autophagy, and modulating immune function. However, further work is needed to fully elucidate the precise molecular mechanisms of Rhap's action and its pharmacokinetic profile to assess its translational potential in humans.

**Supplementary Information:**

The online version contains supplementary material available at 10.1186/s13020-025-01319-3.

## Introduction

Aging is a complex biological process that is characterized by a progressive decline in physiological function and increased susceptibility to age-related diseases [1, 2]. Cellular senescence, which is a state of irreversible cell cycle arrest, is a key hallmark of aging and contributes to tissue dysfunction through the secretion of proinflammatory cytokines, chemokines, and matrix-degrading enzymes; collectively, these phenomena are known as the senescence-associated secretory phenotype (SASP) [3, 4]. The accumulation of senescent cells has been implicated in various age-related conditions, including cardiovascular diseases, neurodegeneration, and metabolic disorders [5–7].

The antiaging therapeutic strategies that are currently available target cellular senescence, reduce systemic inflammation, and promote cellular repair mechanisms [8, 9]. Drugs, such as senolytics, which selectively eliminate senescent cells, have shown promise in extending healthspans in preclinical models [10, 11]. However, the off-target effects and potential toxicity of these compounds limit their clinical applicability [12]. Natural compounds, particularly polyphenols, have emerged as attractive candidates for use in antiaging therapeutic strategies because of their ability to modulate multiple aging-related pathways and relatively low toxicity [13]. Despite these advances, effective and safe antiaging agents that can simultaneously address multiple aspects of aging are needed.

Rhapontigenin (Rhap; 3,3’,5-trihydroxy-4’-methoxy-stilbene, C_15_H_14_O_4_) is a hydroxylated and methoxylated stilbene compound that is derived mainly from the rhizomes of the Chinese herbal medicinal *Rheum rhaponticum* and *Rheum undulatum*, both of which have been extensively utilized in traditional Asian medicine for the treatment of inflammation, digestive disorders, and systemic imbalances [14, 15]. Rhap has been reported to scavenge intracellular reactive oxygen species (ROS), inhibit DNA, RNA, and protein synthesis, and induce the apoptosis of certain pathogens [16, 17]. Moreover, Rhap has diverse pharmacological activities, including antioxidant, anti-inflammatory, anticancer, cardioprotective, lipid-lowering, and antimicrobial activities [18–20]. Given these properties, Rhap has potential for use as an agent to treat oxidative stress, inflammation, and cellular damage, all of which are major contributors to aging.

In this study, we aimed to evaluate the potential of Rhap to be used as an antiaging agent. We investigated the effects of Rhap on cellular senescence, physical performance, immune modulation, and autophagy in both in vitro and in vivo models of aging. Our findings suggest that Rhap may be a promising candidate for mitigating age-related cellular and physiological decline, highlighting its potential for use as a therapeutic agent for age-associated conditions.

## Materials and methods

### Chemicals

Rhapontigenin (Rhap, C_15_H_14_O_4_, purity ≥ 99.92%) was purchased from Selleck (S9163, Selleck, Shanghai, China).Resveratrol (Resv, C_14_H_12_O_3_, purity ≥ 99.99%) was purchased from Selleck (S1396, Selleck, Shanghai, China). Etoposide (Etop, C_29_H_32_O_13_, purity ≥ 99.96%) was purchased from Selleck (S1225, Selleck, Shanghai, China). Hydrogen peroxide (H_2_O_2_) and sodium carboxymethyl cellulose (CMC-NA) were obtained from Sigma‒Aldrich (323,381 and 419,273, Sigma‒Aldrich, Merck, Germany). Dimethyl sulfoxide (DMSO) was purchased from Solarbio (D8371, Solarbio, Beijing, China).

### Cell culture

NIH3T3 cells were authenticated by STR profiling (March 2025) and tested negative for mycoplasma contamination, as certified by Wuhan Pricella Biotechnology Co., Ltd. (Wuhan, China). The cells were cultured in DMEM/F12 medium (CD0001, SparkJade, Wuhan, China) supplemented with 10% fetal bovine serum (FBS, Biological Industries) and 1% penicillin/streptomycin (P/S, 10,378,016, Gibco). IMR90 cells were also purchased from Wuhan Pricella Biotechnology Co., Ltd. and authenticated by STR. They were cultured in Minimum Essential Medium (MEM) supplemented with 10% FBS and 1% P/S. Both cell lines were maintained in a humidified incubator at 37 °C with 5% CO₂.

To induce senescence, NIH3T3 cells were treated with 400 μM H_2_O_2_ for 3 h, washed three times with phosphate-buffered saline (PBS), and then cultured in fresh medium for 24 or 48 h. To evaluate the anti-senescence effects of Rhap, the cells were first treated with 400 μM H_2_O_2_for 3 h, washed with PBS, and incubated overnight in fresh medium. Then, the medium was replaced with medium supplemented with different concentrations of Rhap. IMR90 cells were exposed to 50 μM Etop for 48 h to induce senescence, followed by incubation in normal medium for 3 days. For anti-senescence experiments, IMR90 cells were treated with Etop, and then, the medium was replaced with medium supplemented with different concentrations of Rhap.

### Animals

Male C57BL/6JNifdc mice, aged 19 months (old) or 5 weeks (young), were purchased from Weitong Lihua Experimental Animal Technical Co., Ltd. (Beijing, China). The mice were housed in a specific pathogen-free (SPF) environment at the Experimental Animal Center of Shandong University with a 12:12 light/dark cycle, a temperature of 23 ± 1 °C, a humidity of 40–70%, and the mice were given free access to food and water. After one week of acclimatization, the old mice were randomly assigned to three groups (n = 6/group) and received either vehicle or Rhap (50, 100 mg/kg) via oral gavage every two days for two months. These doses were selected based on the previous literature [41]. Behavioral tests and tissue collection were conducted two weeks after the final gavage. All the animal experiments complied with ARRIVE (Animal Research: Reporting of In Vivo Experiments) guidelines and were performed in accordance with the Guide for the Care and Use of Laboratory Animals (NIH publication 86–23) and approved by the Animal Ethical and Welfare Committee (AEWC) of Shandong University Cheeloo College of Medicine (No. 21172).

### Behavioral tests: grip strength, rotarod, and tail suspension tests

Grip strength was assessed with a Grip Strength Meter (SA417, Sansbio, Jiangsu, China). The mice were lifted by the tail and allowed to grasp a bar with their front paws. Then, the mice were pulled horizontally until they released their grip on the bar. The force exerted was recorded, and three measurements per mouse were averaged.

Motor coordination and balance were evaluated using the rotarod test (LE8205, Panlab Harvard Apparatus). The mice were trained for three days at rotation speeds of 4, 6, and 8 revolutions per minute (rpm) for 200 s in each session. On the test day, the speed of the rod was increased from 4 to 40 rpm over the course of five minutes. Each mouse was subjected to the test three times, and the average latency to fall and rotation speed were recorded.

The tail suspension test (TST) was used to assess depressive-like behavior. The mice were suspended by the tail from a hook at the top of a wooden frame, 50 cm above the floor, for six minutes. Mouse behavior was recorded and analyzed with TopScan software. The immobility time was defined as the period during which the mice remained motionless and did not actively attempt to escape.

### Senescence-associated β-galactosidase (SA-β-gal) staining

SA-β-Gal activity was measured with a Senescence β-Galactosidase Staining Kit (C0602, Beyotime, Shanghai, China) according to the manufacturer's protocol. Cells or tissue sections were fixed with β-Gal staining fixative for 15 min at room temperature (RT), washed with PBS, and then incubated with β-Gal staining solution for 16–18 h at 37 °C in a CO₂-free incubator. Stained cells or sections were observed under a light microscope, and the number of SA-β-Gal-positive cells or area of positive staining was quantified using ImageJ software.

### Western blotting

Western blotting was performed as previously described [21]. Cultured cells were lysed with RIPA buffer (PC101, Epizyme, Shanghai, China) supplemented with protease and phosphatase inhibitors (78,442, Thermo Fisher Scientific, MA, USA). The protein concentrations were measured with a BCA Protein Assay Kit (23,227, Thermo). Equal amounts of proteins were subjected to SDS‒PAGE and transferred to PVDF membranes (ISEQ00010, Millipore, USA). After being blocked with Protein-Free Rapid Blocking Buffer (PS108, EpiZyme, Shanghai, China), the membranes were incubated overnight at 4 °C with primary antibodies, followed by incubation with secondary antibodies for 1 h at RT. The protein bands were visualized using Immobilon Western HRP Substrate (WBKLS0500, Millipore) and quantified using Image Lab Software (Bio-Rad) and ImageJ. The primary antibodies used are as follows: anti-p16^ink4a^ antibody (1:1000, ab211542, Abcam), anti-p21^cip1^ antibody (1:1000, ab188224, Abcam), anti-Sirt1 antiboody (1:1000, ab110304, Abcam), anti-p-AMPK antibody (1:2000, ab133448, Abcam), anti-AMPK antibody (1:1000, ab207442, Abcam), anti-p62 antibody (1:1000, 5114S, CST), anti-LC3B antibody (1:1000, NB100-2220SS, Novus), anti-Beta Actin antibody (1:10,000, 20536-1-AP, Proteintech).

### Hematoxylin and eosin (H&E) staining

H&E staining was performed as previously described [22]. Liver tissues were dissected, fixed in 4% paraformaldehyde for 48 h, processed in an embedding box, and embedded in paraffin. Sections (5 μm) were cut and stained with an H&E Stain Kit (G1120, Solarbio) according to the manufacturer's instructions. Stained sections were examined under a light microscope, and pathology was assessed by a blinded veterinary pathologist.

### Oil Red O staining

Lipid accumulation in liver tissues was assessed with Oil Red O staining. Liver tissues were dissected, washed with PBS, embedded in optimal cutting temperature (OCT) compound, and frozen at − 80 °C. Frozen Sects. (10 μm) were stained with an Oil Red O Stain Kit (ab150678, Abcam) according to the manufacturer's instructions. The relative lipid content in 10 random fields from three mice was quantified using ImageJ.

### Masson staining

To assess liver fibrosis, Masson's trichrome staining was performed with a Masson’s Trichrome Stain Kit (G1340, Solarbio) according to the manufacturer's instructions. Paraffin-embedded sections were dewaxed, rehydrated, and stained with Weigert’s iron hematoxylin, acid fuchsin, phosphomolybdic acid, and aniline blue. Fibrosis was quantified as the ratio of the blue collagen-positive area to the red normal area.

### Quantitative polymerase chain reaction (qPCR)

qPCR was performed as previously described [23], with slight modifications. Total RNA was extracted from frozen spleen and liver tissues using TRIzol Reagent (15,596,026, Invitrogen). cDNA synthesis was carried out using the SPARKscript II RT Plus Kit (AG0304, SparkJade). qPCR was performed using 2 × SYBR Green qPCR Mix (AH0101, SparkJade) on a Roche LightCycler 480 instrument. Actin-β was used as the internal reference gene, and the comparative quantification method was 2^(− ΔΔCt). The sequences of the primers that were used are listed in Supplementary Table S1.

### Immunofluorescence analysis

Cryosections of liver and spleen tissues were incubated in PBS for 10 min to remove the OCT compound. Antigens were retrieved by boiling and heating for 10 min in 1 × Tris–EDTA antigen repair solution (pH 9.0, C1038, Solarbio). The sections were then permeabilized with 0.5% Triton X-100 for 20 min at RT. After being blocked with QuickBlock Blocking Buffer for Immunol Staining (P0260, Beyotime), the sections were incubated in a wet box with primary antibodies against p21 (1:200, ab188224, Abcam), p16 (1:200, ab211542, Abcam) or Sirt1 (1:200, ab110304, Abcam) at 4 °C overnight. Then, the sections were washed three times in PBS, followed by incubation with the Alexa Fluor 488-conjugated goat anti-rabbit IgG H&L (1:500, ab150077, Abcam), Alexa Fluor 594-conjugated goat anti-rabbit IgG H&L (1:500, ab150080, Abcam) or Alexa Fluor 594-conjugated goat anti-mouse IgG H&L (1:500, ab150116, Abcam) secondary antibodies for 2 h at RT in the wet box in the dark. After being washed with PBS, the sections were mounted with Mounting Medium, antifading (with DAPI) (S2110, Solarbio). Finally, images of randomly selected fields were captured at 20 × magnification by fluorescence microscopy (Olympus BX53). Images were randomly captured of 3 fields per mouse from 3 mice. The fluorescence intensity was analyzed with ImageJ.

### Flow cytometry analysis

The spleen and peripheral blood were harvested from mice, and single-cell suspensions were prepared according to previously described methods [24]. Blood was collected from anesthetized mice by heart puncture and transferred to EDTA-coated tubes. Moreover, spleens were harvested from euthanized mice and placed in cold PBS. After the spleens were pierced with 1-ml syringes, spleen cells were removed with 1 × PBS and filtered through a 70-μm cell strainer. Red blood cells in the peripheral blood and splenocytes samples were lysed using ACK lysis buffer (Cat. No. A1049201, Gibco) according to the manufacturer’s instructions. Then, the cells were washed with cold stain buffer (FBS) (BD Biosciences, Cat No. 554656) and incubated with an anti-mouse CD16/32 mAb (BD Biosciences, 553,141) for 5 min on ice to block the Fc receptor. The cells were subsequently incubated with fluorescently labeled primary antibodies for 30 min on ice in the dark. The antibodies that were used to stain the blood or spleen single-cell suspensions were as follows: PE-conjugated anti-CD3 (Clone No. 145-2C11, 1:100, BD Biosciences, Cat No. 553063), PerCP-conjugated anti-CD4 (RM4-5, 1:100, BD Biosciences, 553,052), BV421-conjugated anti-CD8 (53–6.7, 1:100, eBioscience, 48–0081-82), FITC-conjugated anti-NK1.1 (PK136, 1:100, BD Biosciences, 553,164). After staining, the cells were washed, resuspended in staining buffer and detected with a BD LSRFortessa flow cytometer. Data analysis was performed with FlowJo (v10.7.2).

### Clinical chemistries

At the end of the experiments with aged mice, the mice were anesthetized with isoflurane, and blood was collected into EDTA anticoagulation tubes. Plasma was obtained by centrifugation (3000 rpm) at 4 °C for 15 min, and then, it was aliquoted and stored at − 80 °C until analysis. The concentrations of plasma biochemical parameters, such as ALT, AST, CHE, UREA, CREA, and UA, were measured by a fully automated biochemical analyzer according to the manufacturer’s instructions. Plasma samples from 5 mice per group were analyzed.

### Luminex

Plasma samples were collected and stored as described above. The levels of cytokines in the plasma samples were measured on a Luminex X-200 instrument with a Bio-Plex Pro Mouse Cytokine 23-plex Assay (M60009RDPD, Bio-Rad) according to the manufacturer’s instructions.

### Cell counting kit-8 assay

Cell viability was determined with a Cell Counting Kit-8 (CCK8, Dojindo, Japan). A total of 1 × 10^4^ cells were seeded in each well of a 96-well plate and treated with vehicle (DMSO) or 5, 10, 15, or 20 μM Rhap for 24 h after the cells had attached to the wells. After washing with PBS, 100 µl of medium supplemented with 10 µl of CCK8 reagent was added. The absorbance at 450 nm was measured after 2 h of incubation in a cell culture incubator. To calculate the half maximal inhibitory concentration (*IC*_*50*_), 5 × 10^3^ cells were seeded in each well of a 96-well plate and treated with vehicle (DMSO) or 0.01, 0.1, 1, 10, 50, 100, 500, 1000 μM Rhap for 48 h after the cells had attached to the wells. The absorbance at 450 nm was measured after 1 h of incubation with CCK8 reagent. *IC*_*50*_ was determined by fitting the concentration–response data to a nonlinear regression model using GraphPad Prism software (GraphPad Software, San Diego, CA, USA).

### EdU cell proliferation assay

A total of 1 × 10^4^ NIH3T3 cells were seeded in each well of 96-well plates and treated with (5, 10, 15, or 20 μM) or without Rhap for 24 h after the cells had attached to the wells. EdU-based cell proliferation was measured with a Cell-LightTM EdU Apollo In Vitro Kit (C10310-3, RiboBio) following the manufacturer's instructions. EdU signals were measured by flow cytometry, and the results were analyzed with FlowJo.

### Molecular docking

Molecular docking analysis was conducted using AutoDock Vina [25]. The 3D protein structure of Sirt1 (PDB ID: 4if6, 4kxq, 4i5i and 4zzj) was downloaded from the RCSB PDB website (https://www.rcsb.org/), and the compound structure of Rhap (PubChem CID: 5,320,954) was obtained from PubChem (http://pubchem.ncbi.nlm.nih.gov/). The binding affinity was used to predict the optimal binding between the compound and the protein.

3D structural diagrams showing the interaction between the compound and protein were generated using PyMol software. The binding sites were identified on the PLIP website (http://projects.biotec.tu-dresden.de/plip-web/plip/index) [26] and visualized using PyMol software.

### Microscale thermophoresis (MST)

To determine the binding affinity between SIRT1 and Rhapontigenin, MST analysis was performed using a Monolith NT.115 instrument (Zoonbio Biotechnology Co., Ltd., Nanjing, China). Purified SIRT1 protein was fluorescently labeled with the RED-NHS 2nd Generation Kit (MO-L011, NanoTemper Technologies, Munich, Germany) according to the manufacturer's protocol, yielding a final concentration of 0.1 µM. For ligand dilution, Rhapontigenin was serially diluted twofold in PBST buffer containing 5% DMSO, spanning a concentration range from 0.25 mM to 7.63 nM (to avoid precipitation at high concentrations). For binding measurements, 10 µL of gradient-diluted ligand was mixed with 10 µL of labeled SIRT1 and incubated for 5 min at room temperature. Samples were loaded into standard capillaries (MO-K022) and analyzed under optimized MST conditions. Data processing was performed using MO. Control software to calculate the dissociation constant (K_d_) with signal-to-noise ratios maintained at > 5 to ensure reliability.

### Transmission electron microscopy (TEM)

Autophagosomes and autolysosomes in the cells were observed and photographed by TEM, which was performed by Wuhan Servicebio Technology Co., Ltd. In brief, NIH3T3 cells were collected after they were treated with or without 400 μM H_2_O_2_ for 3 h and then cultured in medium supplemented with or without 20 μM Rhap for 48 h. Then, cell pellets were fixed for 2 h in electron microscope fixative supplemented with 2.5% glutaraldehyde (G1102, Servicebio). After being washed three times with 0.1 M phosphate buffer (pH 7.4), the cell pellets were encapsulated in 1% agarose and then fixed with 1% osmic acid in 0.1 M phosphate buffer (pH 7.4) for 2 h at room temperature in the dark. The samples were dehydrated with gradient alcohol and acetone. The samples were subsequently infiltrated and embedded with 812 embedding agent before being allowed to polymerize in an oven at 60 °C for 48 h. The resin blocks were sectioned at a thickness of 60 nm with an ultrathin microtome, and the pieces were harvested with 150-mesh square Chinese membrane copper mesh. Then, ultrathin sections in the copper mesh were stained with 2% uranyl acetate saturated with alcohol solution and 2.6% lead citrate. The samples were subsequently observed and imaged by TEM (Hitachi HT7800). Finally, the numbers of autophagic vacuoles (AVs, including both autophagosomes and autolysosomes) were counted and analyzed.

### RNA-seq and data analysis

Total RNA was isolated from snap-frozen liver tissues with a TRIzol RNA extraction kit (TIANGEN, Cat. No. DP424), yielding > 2 μg of total RNA per sample. The RNA quality was examined by 0.8% agarose gel electrophoresis and spectrophotometry, and high-quality RNA with a 260/280 ratio ranging from 1.8 to 2.2 was used for library construction and sequencing. The library was constructed following the Illumina manufacturer's instructions (Illumina, USA). Oligo-dT primers were utilized to transcribe mRNA into cDNA (APExBIO, Cat. No. K1159). cDNA was amplified for the synthesis of the second chain of cDNA. The cDNA products were purified with the AMPure XP system (Beckman Coulter, Beverly, USA). After library construction, the library fragments underwent enrichment by PCR amplification and were selected on the basis of a fragment size of 350–550 bp. Next, the library was quality-assessed with an Agilent 2100 Bioanalyzer (Agilent, USA). Then, the library was sequenced on the Illumina NovaSeq 6000 platform (paired-end 150) to produce raw reads. Raw data have been deposited to National Center for Biotechnology Information (NCBI) under the BioProject number PRJNA1235652.

Raw paired-end fastq reads were filtered by TrimGalore to discard the adapters and low-quality bases via calling the Cutadapt tool. The obtained clean reads were subsequently aligned to the mm10 mouse genome using HISAT2 [27], followed by reference genome-guided transcriptome assembly and gene expression quantification with StringTie [28]. Differentially expressed genes (DEGs) of samples with replications were identified using DEseq2 [29] or edgeR [30] for samples with no replication, with thresholds of |fold-change|> 1.5 and *p* < 0.05. The functional enrichment analysis of the annotated significant DEGs and the potential genes in the identified modules based on Gene Ontology (GO) and KEGG pathway categories was performed using the clusterProfiler [31]. Terms with *p* values < 0.05 were considered significant.

### Cell transfection with siRNAs

The mouse negative control siRNA (si-NC) and siRNA targeting Sirt1 (si-Sirt1) were purchased from GeneChem (Shanghai, China). NIH3T3 cells were transfected with siRNAs using LipofectamineTM 2000 (11,668,019, Invitrogen, CA, USA) following the manufacturer’s instructions. We used two si-Sirt1 sequences to prevent off-target effects. The sequences of the si-Sirt1 that were used were as follows: si-Sirt1-1 sense 5’-GCCAUGUUUGAUAUUGAGUAUdTdT-3’ and antisense 5’-AUACUCAAUAUCAAACAUGGCdTdT-3’; si-Sirt1-2 sense 5’-AGUGAGACCAGUAGCACUAAUdTdT-3’ and antisense 5’-AUUAGUGCUACUGGUCUCACUdTdT-3’.

### Statistical analysis

All the experimental data are presented as the mean ± standard deviation (SD). Statistical analyses were conducted using GraphPad Prism 8.0 software. Comparisons between two groups were performed using Student's t test, whereas comparisons among multiple groups were performed using one-way analysis of variance (ANOVA) followed by Tukey's post hoc test. The significance level was set to *p* < 0.05. *C. elegans* survival analyses were conducted using the log rank (Mantel‒Cox) test.

## Results

### Rhap reduces oxidative stress-induced cellular senescence in vitro

**Fig. 1:**
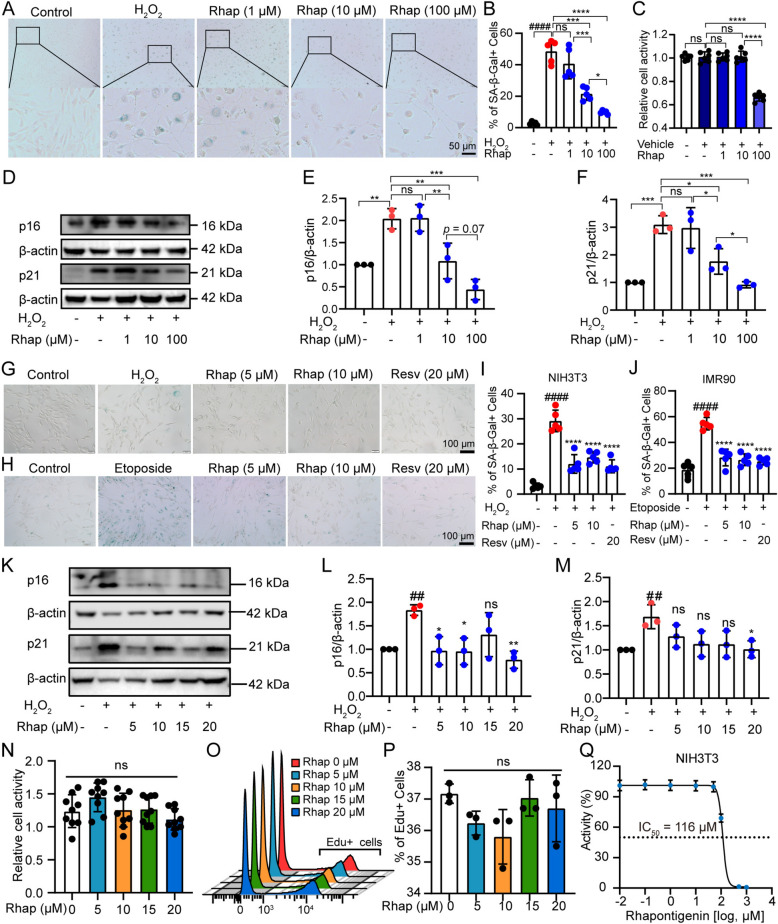
Rhapontigenin (Rhap) attenuates cellular senescence in vitro. **A** NIH3T3 cells were treated with 400 μM H₂O₂ for 3 h and then with Rhap (1, 10, and 100 μM) for 48 h, followed by SA-β-Gal staining. **B** Quantification of SA-β-Gal-positive cells from (**A**). **C** Viability of NIH3T3 cells treated with various Rhap concentrations was assessed with the CCK-8 assay. **D**–**F** Western blotting analysis of p16 and p21 expression in NIH3T3 cells treated with H_2_O_2_ and Rhap. β-actin served as the loading control. **G**, **I** NIH3T3 cells treated with 400 μM H2O2 for 3 h followed by Rhap (5 or 10 μM) for 48 h exhibited reduced senescence, as indicated by SA-β-Gal staining. Resveratrol (Resv, 20 μM) was used as a positive control of efficacy. **H**, **J** IMR90 cells treated with 50 μM etoposide for 48 h followed by Rhap (5 or 10 μM) for 72 h exhibited decreased senescence. Resv (20 μM) was used as a positive control of efficacy. **K**–**M** Western blotting analysis of p16 and p21 expression in NIH3T3 cells treated with H_2_O_2_ and Rhap. **N** Viability of NIH3T3 cells treated with various Rhap concentrations was assessed with the CCK-8 assay. **O**, **P** EdU staining and flow cytometry revealed that Rhap had no significant effect on NIH3T3 cell proliferation. **Q** IC50 of Rhap in NIH3T3 cells after 48 h treatment (CCK-8 assay, nonlinear regression fit). The data are presented as the means ± SDs. Statistical analysis was performed with one-way ANOVA with Tukey's multiple comparisons. ## *p* < 0.01 versus the Control group, #### *p* < 0.0001 versus the Control group, * *p* < 0.05 versus the H_2_O_2_ group, ** *p* < 0.01 versus the H_2_O_2_ group, **** *p* < 0.0001 versus the H_2_O_2_ group, ns = not significant

To determine the effect of Rhap on cellular senescence, NIH3T3 and IMR90 cells were subjected to oxidative or genotoxic stress, followed by treatment with Rhap. Supplementary Figure S1 shows that NIH3T3 cells treated with increasing concentrations of H₂O₂ exhibited a dose-dependent increase in senescence, as indicated by SA-β-Gal staining and increased expression of the senescence markers p16 and p21 (Fig. S1A-E). Based on these results, an H₂O₂-induced senescence model was established in NIH3T3 cells using 400 μM H₂O₂. In this model, treatment with 10 and 100 μM Rhap significantly reduced the number of senescent cells (Fig. 1A, B) and downregulated the expression of senescence-associated markers p16 and p21 (Fig. 1D–F). However, 100 μM Rhap markedly reduced normal cell viability (Fig. 1C). Based on these preliminary results and previously reported effective concentrations of analogous compounds [32], we selected a concentration range of 5 to 20 μM for further investigation.

Subsequent treatment with Rhap (5 or 10 μM) significantly reduced the number of senescent cells (Fig. 1G, I). Similarly, in IMR90 cells, Etop-induced senescence was attenuated by Rhap treatment (Fig. 1H, J). Western blotting analysis further demonstrated that Rhap treatment reduced the expression of the senescence-associated markers p16 and p21 in NIH3T3 cells (Fig. 1K–M). These findings suggest that Rhap effectively mitigates oxidative and genotoxic stress-induced cellular senescence. The CCK-8 assay showed that treatment with Rhap at concentrations ranging from 5 to 20 μM did not significantly reduce cell viability, indicating a lack of cytotoxicity at these doses (Fig. 1N). Moreover, EdU staining and flow cytometry revealed that Rhap did not impair cell proliferation, indicating that its anti-senescence effects did not occur due to reduced cell viability or proliferation (Fig. 1O, P). The 50% inhibitory concentration (*IC*_*50*_) of Rhap on NIH3T3 cell viability after 48 h was 116 μM (*95*% *CI*, 107.9–124.8 μM). Furthermore, Rhap did not affect cell viability at concentrations up to 50 μM, which aligns with our previous observations (Fig. 1Q).

In addition, the impact of Rhap on organismal longevity was evaluated with N2 nematodes. Survival analysis revealed a significant extension in lifespan after Rhap treatment. Compared to the vehicle group, the median lifespan of nematodes treated with 0.2 mM Rhap was extended by 21.7% and the maximum lifespan by 15%; the median lifespan of nematodes treated with 1 mM Rhap was extended by 30.4% and the maximum lifespan by 20%. (Fig. S2 and Table S2). The results suggests that the potential benefits of Rhap beyond those observed in cellular models.

### Rhap increases physical performance and maintains organ function in aged mice

**Fig. 2:**
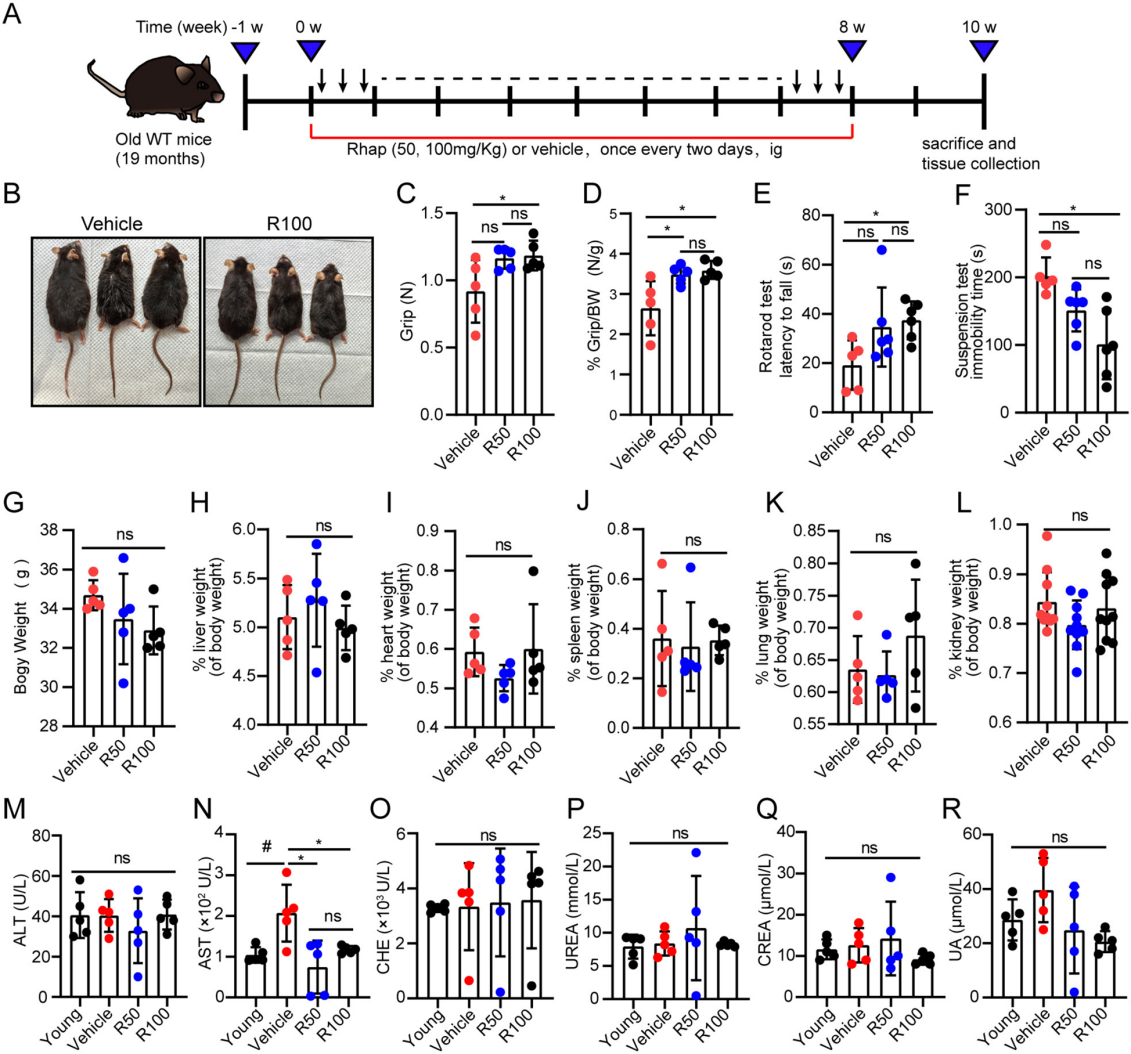
Rhapontigenin (Rhap) increases physical performance and organ health in aged mice. **A** Schematic representation of the experimental design. Aged mice (19 months) were treated with 50, 100 mg/kg Rhap or vehicle every two days for 8 weeks, followed by tissue collection at 10 weeks. **B** Representative images of aged mice treated with vehicle or Rhap. **C** Grip strength analysis. **D** Grip strength normalized to body weight. **E** Rotarod performance. **F** Tail suspension test results. **G**–**L** Body and organ weights, including liver, heart, spleen, lung, and kidney weights. **M**–**R** Plasma biochemical parameters, including ALT, AST, CHE, UREA, CREA, and UA. The data are presented as the means ± SDs. Statistical analysis was performed with one-way ANOVA with Tukey's multiple comparisons or Student's t test. ### *p* < 0.001 versus the Young group, * *p* < 0.05 versus the Vehicle group, ** *p* < 0.01 versus the Vehicle group, *** *p* < 0.001 versus the Vehicle group, ns = not significant. R50, Rhapontigenin 50 mg/kg; R100, Rhapontigenin 100 mg/kg; ALT, alanine aminotransferase; AST, aspartate transaminase; CHE, cholinesterase; CREA, creatinine; UA, uric acid

To evaluate the effects of Rhap on aging, 19-month-old mice were treated with Rhap or vehicle for 8 weeks (Fig. 2A). The mice treated with Rhap had smoother fur than the control group (Fig. 2B). Rhap-treated mice exhibited significantly improved physical performance, as indicated by increased grip strength normalized to body weight (Fig. 2C, D) and enhanced motor coordination in the rotarod test (Fig. 2E). Furthermore, Rhap-treated mice demonstrated shorter immobility times in the TST, suggesting improved mood or reduced depressive-like behavior (Fig. 2F).

**Fig. 3:**
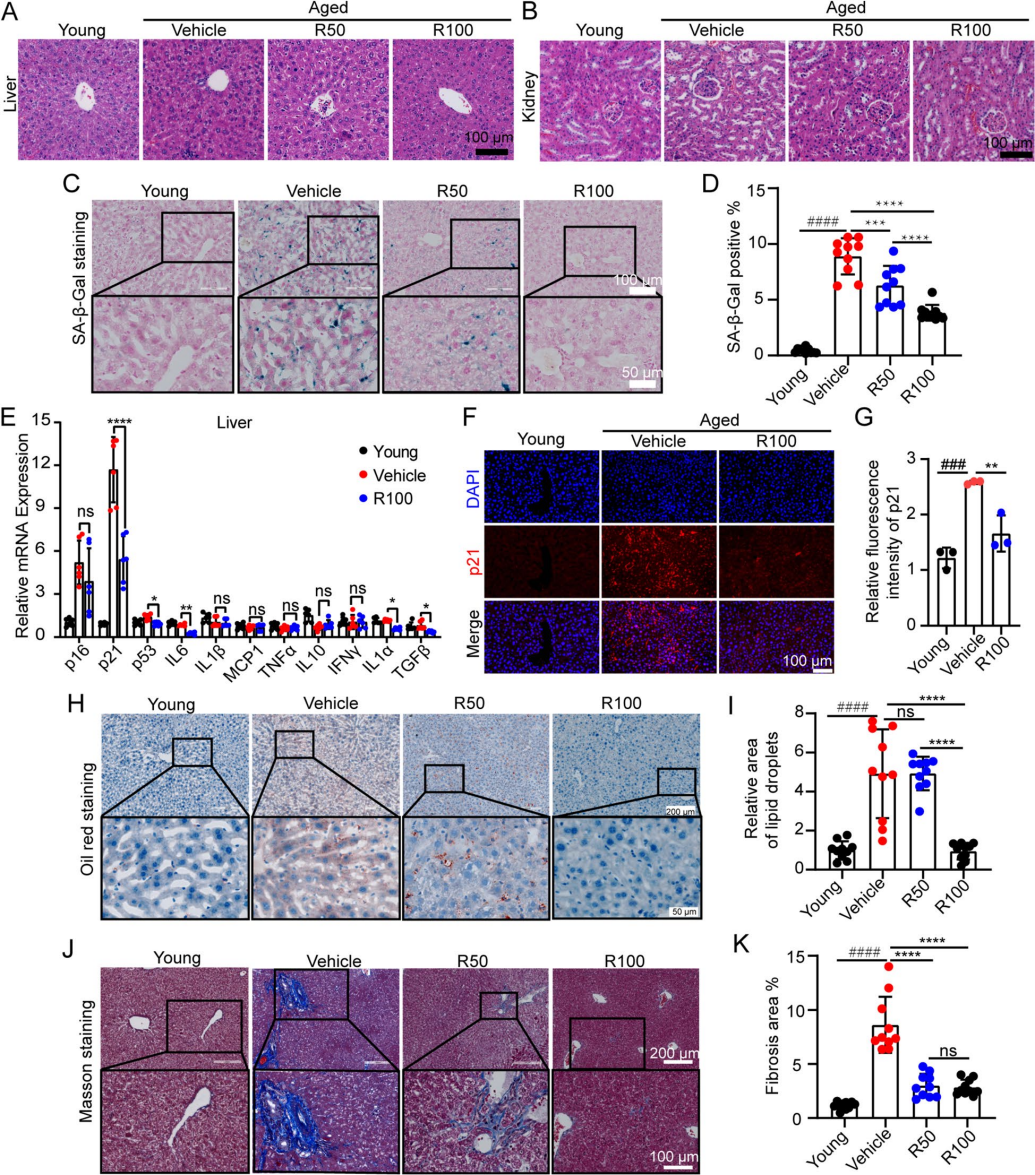
Administration of Rhap attenuates liver senescence in aged mice. **A** HE staining was used to assess pathological features of liver tissues (scale bar, 100 μm). **B** Pathological characteristics of kidney tissues as assessed by HE staining (scale bar, 100 μm). **C** SA-β-Gal staining in liver sections from young mice and old mice treated with vehicle or Rhap (20 × magnification). **D** Percentage of the total area that was positive for SA-β-Gal staining in (**C**). **E** The transcript levels of the senescence-associated markers p16, p21, and p53 and the SASP factors IL-6, IL1-β, MCP-1, TNFα, IL-10, IFNγ, IL-1α, and TGF-β in the livers of the mice were measured by qPCR. **F** Representative immunofluorescence images of immunofluorescence staining for p21 in liver tissues (scale bar = 100 μm). **G** Quantification of the fluorescence intensities of p21. **H** Representative images of Oil Red O-stained liver sections (10 × original magnification). **I** Quantification of the Oil Red O-positive area. **J** Representative images of Masson-stained liver tissues (10 × original magnification). **K** Quantification of the fibrotic area as a percentage of the entire field of view. n = 10 fields from 3 mice. The data are presented as the means ± SDs, n = 3–5 mice/group. Statistical analysis was performed with Student’s t test or one-way ANOVA. #### *p* < 0.0001 versus the Young group, * *p* < 0.05 versus the Vehicle group, ** *p* < 0.01 versus the Vehicle group, **** *p* < 0.0001 versus the Vehicle group, ns, no significance. SASP, senescence-associated secretory phenotype

Rhap treatment did not significantly affect body weight or organ weights (liver, heart, spleen, lung, and kidney weights), indicating that it did not have adverse effects on overall health (Fig. 2G–L). Plasma biochemical analysis revealed a significant reduction in AST levels in Rhap-treated mice, suggesting improved liver function, whereas other markers (ALT, CHE, UREA and CREA) remained unchanged (Fig. 2M–R). Histological analysis by HE staining revealed no adverse effects in liver and kidney pathology due to Rhap treatment (Fig. 3A, B). These results indicate that Rhap increases physical function and maintains organ health in aged mice and lacks liver or kidney toxicity at the concentrations used, highlighting its potential to mitigate age-related declines.

### Rhap attenuates liver senescence, lipid accumulation, and fibrosis in aged mice

To assess the impact of Rhap on aging, SA-β-Gal staining was performed on the tissues of the heart, liver, spleen, lung and kidney. There were no significant differences observed in the tissues of the heart, lungs and kidneys (data not shown). In particular, SA-β-Gal staining revealed a significant increase in the number of senescent cells in the livers of vehicle-treated aged mice, and this effect was markedly suppressed by Rhap treatment (Fig. 3C, D). qPCR analysis revealed that Rhap reduced the expression of senescence markers (p21 and p53) and SASP factors (Fig. 3E). Immunofluorescence staining confirmed that p21 expression was significantly decreased in the livers of Rhap-treated aged mice (Fig. 3F, G).

Oil red O staining revealed reduced lipid accumulation in the livers of Rhap-treated aged mice, suggesting improved lipid metabolism (Fig. 3H, I). Additionally, Masson staining revealed a significant reduction in liver fibrosis in Rhap-treated aged mice compared with control mice (Fig. 3J, K). These findings indicate that Rhap effectively attenuates liver senescence, lipid accumulation, and fibrosis in aged mice, highlighting its potential for use as an agent for treating age-related liver dysfunction.

### Rhap attenuates spleen senescence and modulates immune function in aged mice

**Fig. 4:**
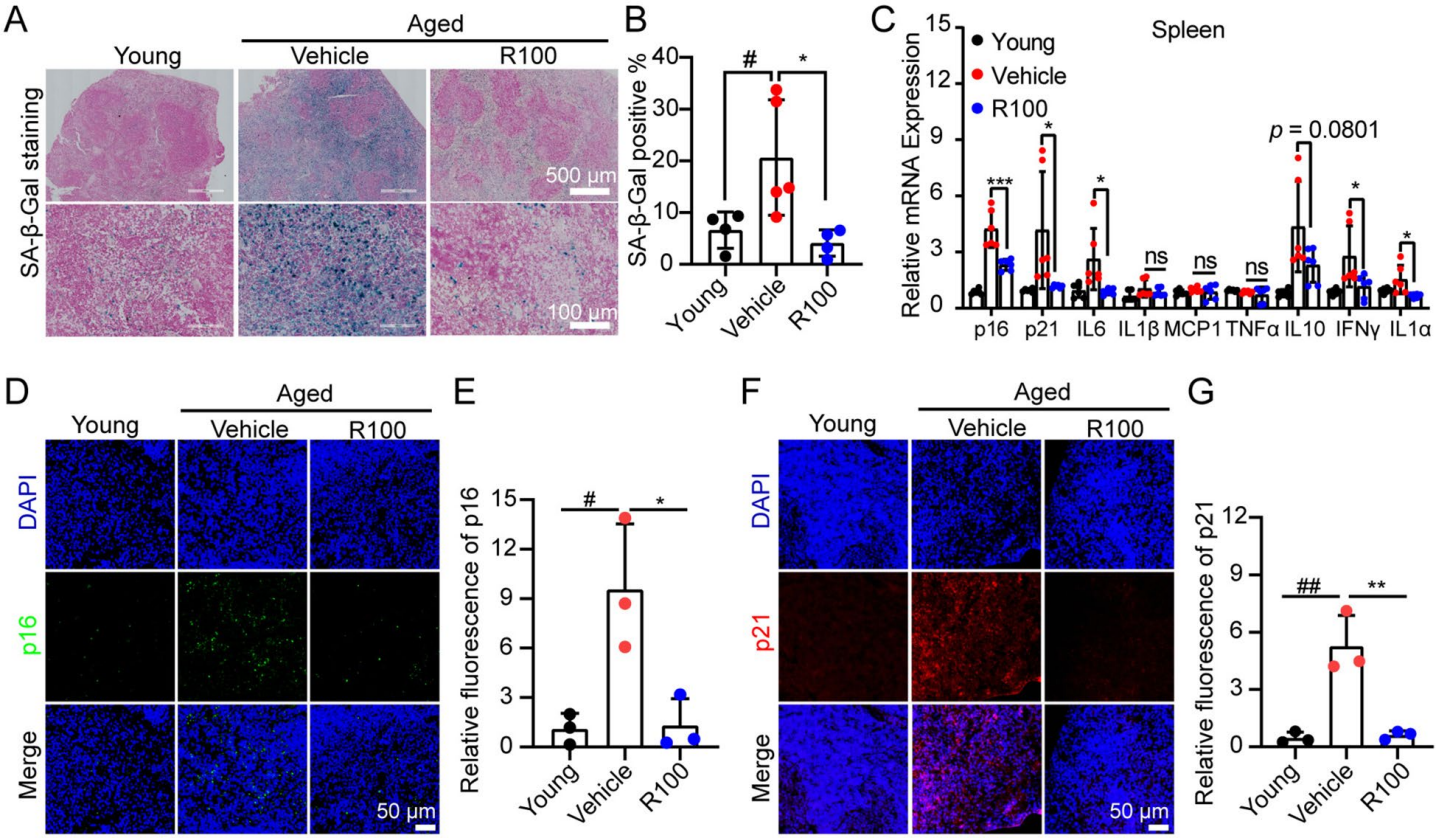
Administration of Rhap reduces the levels of senescence-related markers in the spleens of aged mice. **A** SA-β-Gal staining of spleen sections from young mice and aged mice treated with vehicle or Rhap (5 × or 20 × magnification). **B** Percentage of the total area that was positive for SA-β-Gal staining in (**A**). **C** The transcript levels of senescence-associated markers and SASP factors in the spleens of the mice were measured by qPCR. **D**, **F** Representative images of immunofluorescence staining for p16 and p21 in spleen tissues (scale bar = 50 μm). **E**, **G** Quantification of the fluorescence intensities of p16 and p21 in (**D**, **F**) respectively. The data are presented as the means ± SEM, n = 3–5 mice/group. Statistical analysis was performed with Student’s t test for comparisons between two groups and one-way ANOVA for comparisons among three groups. # *p* < 0.05 versus the Young group, * *p* < 0.05 versus the Vehicle group, ** *p* < 0.01 versus the Vehicle group, *** *p* < 0.001 versus the Vehicle group, ns, no significance

Among the tissues assessed, spleen aging also displayed marked changes following Rhap treatment. Specifically, SA-β-Gal staining revealed a significant decrease in the numbers of senescent cells in the spleens of Rhap-treated aged mice compared with those in the spleens of vehicle-treated control mice (Fig. 4A, B). qPCR analysis revealed that Rhap treatment significantly decreased the expression of senescence markers (p16 and p21) and SASP factors (Fig. 4C).

**Fig. 5:**
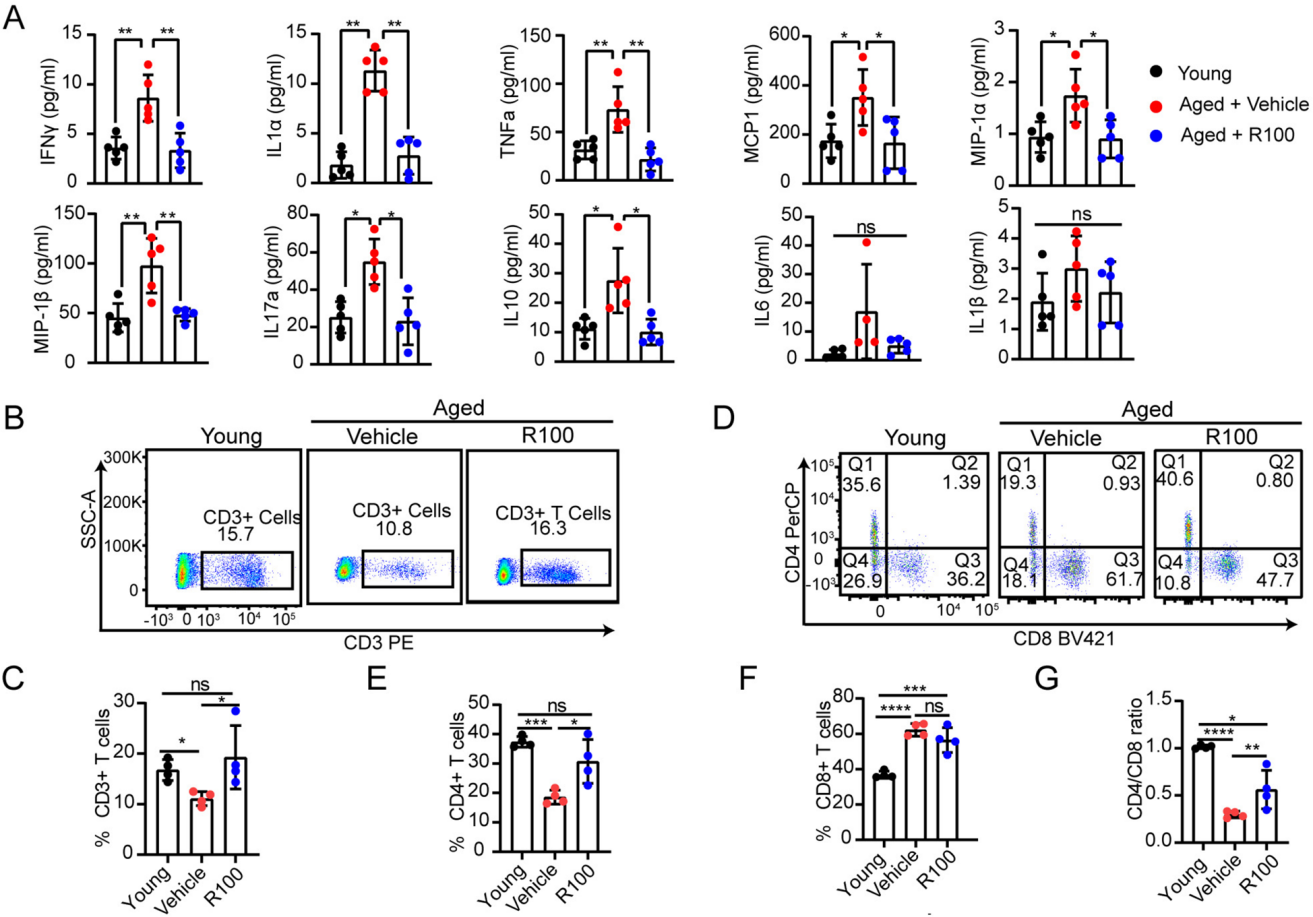
Rhap affects immune function. **A** Cytokine levels in plasma samples from young and aged mice treated with vehicle or Rhap were measured by Luminex. Bar charts showing the plasma cytokine concentrations. **B**, **C** Representative flow cytometric plots and quantification of the proportion of CD3 + T cells in blood samples from the mice. **D**–**G** Representative flow cytometric plots (**D**) and quantification of the proportions of CD4 + T cells (**E**) and CD8 + T cells (**F**) and the ratio of CD4 + T cells/CD8 + T cells (**G**) in blood samples from the mice. The data are presented as the means ± SEM, n = 3–5 mice/group. Statistical analysis was performed with one-way ANOVA. * *p* < 0.05, ** *p* < 0.01, *** *p* < 0.001, **** *p* < 0.0001, ns, not significant. BM, Bone Marrow

Immunofluorescence staining for p16 and p21 further confirmed that expression of these markers was reduced in the spleens of Rhap-treated aged mice (Fig. 4D–G). Moreover, Luminex analysis of plasma samples revealed that compared with vehicle, Rhap significantly reduced the levels of proinflammatory cytokines, including IFNγ, TNFα, IL-1α, IL-17a, and MCP-1, in aged mice, indicating that Rhap exerts an anti-inflammatory effect (Fig. 5A).

To evaluate the effects of Rhap on the immune function, the immune cell subsets were detected using flow cytometry. This analysis revealed that compared with vehicle treatment, Rhap treatment increased the proportion of CD3 + T cells in aged mice (Fig. 5B, C). Furthermore, Rhap treatment significantly increased the proportion of CD4 + T cells, whereas the proportion of CD8 + T cells was not significantly different; these effects resulted in an elevated CD4 +/CD8 + T-cell ratio, which suggests a better T-cell balance (Fig. 5D–G).

It has been shown that mature NK cells are reduced in the peripheral tissues of aged mice compared with young mice [33]. In our study, we found that the proportion of NK cells (NK1.1 + CD3−) in peripheral blood and spleen of aged mice was lower than that of young mice, but there was a trend of increase in Rhap-treated aged mice compared with vehicle-treated group, but the difference was not statistically significant, indicating that Rhap may have an effect on NK cells (Fig. S3A-D). These findings indicate that Rhap effectively reduces splenic senescence and modulates immune function, promoting an anti-inflammatory immune profile in aged mice.

### Rhap upregulates sirt1 expression and promotes autophagy in the liver tissue of aged mice

**Fig. 6:**
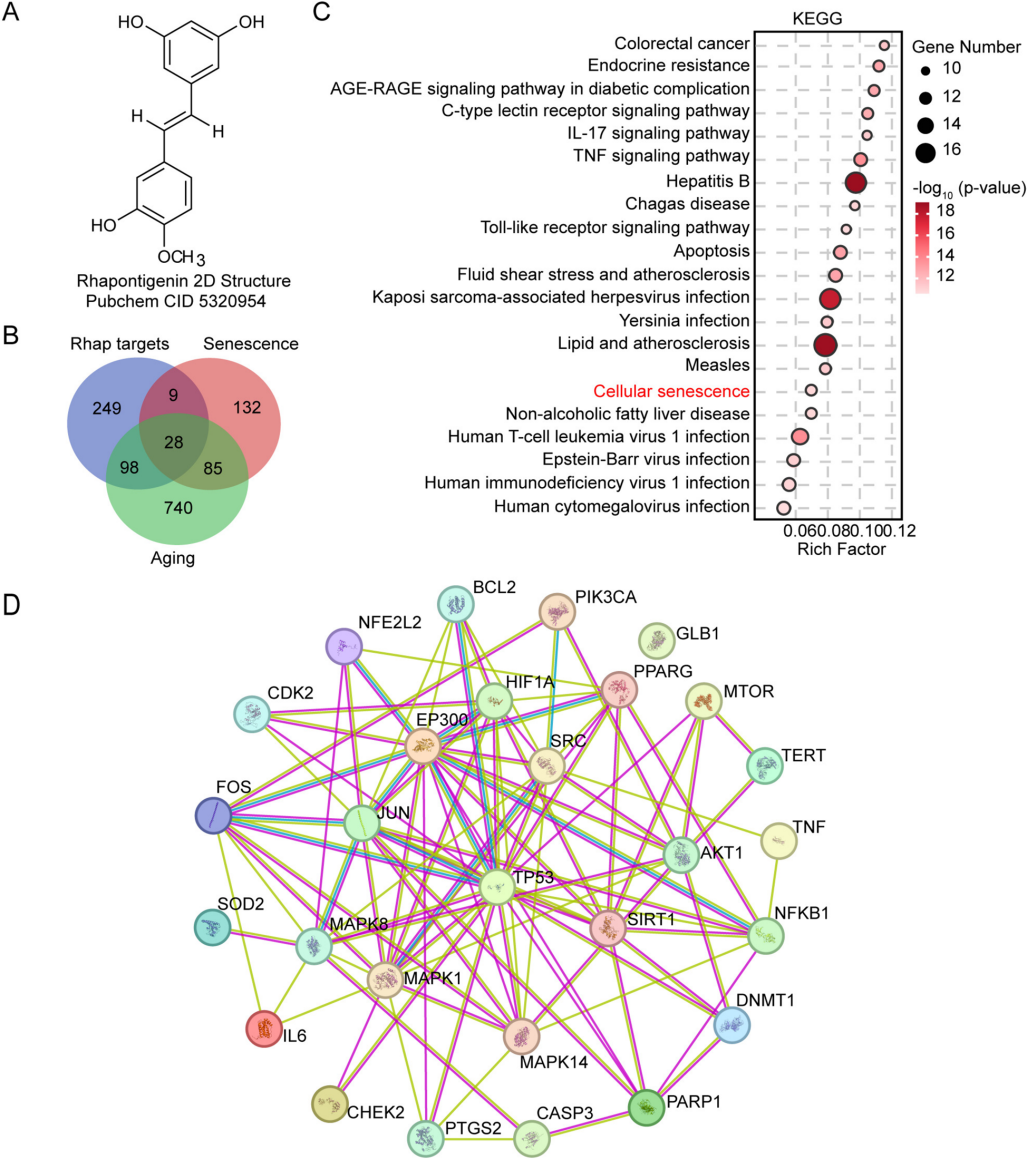
Network pharmacology analysis identifies that Rhapontigenin targets genes related to senescence and aging. **A** Chemical structure of Rhap (PubChem CID 5320954). **B** Venn diagram of senescence targets (relevance score > 3.0, from GeneCards), aging targets (relevance score > 7.0, from GeneCards) and Rhap targets (from the traditional Chinese medicine databases TCMSP, ETCM, BATMAN-TCM, HERB, PubChem and the compound-target prediction websites STITCH, SEA, TargetNet, PharmMapper). **C** KEGG enrichment pathways of shared targets between Rhap, senescence and aging. **D** Construction of the PPI network for shared targets using STRING

Rhap (3,3’,5-trihydroxy-4’-methoxy-stilbene, C_15_H_14_O_4_) is a naturally occurring stilbenoid compound that is characterized by its unique chemical structure featuring a trans-stilbene backbone with hydroxyl groups at the 3, 3’, 5 positions and a methoxy group at the 4’ position (Fig. 6A). Methoxylated stilbenes have been suggested to be more suitable options for oral administration than hydroxylated stilbenes, such as resveratrol, because methoxylated stilbenes have increased bioavailability and similar biological activities compared with hydroxylated stilbenes [34]. To elucidate the molecular mechanisms underlying the antiaging effects of Rhap, we performed a comprehensive analysis of its targets and their roles in aging and senescence-related pathways. A Venn diagram analysis identified 28 genes as shared targets of Rhap, senescence, and aging (Fig. 6B). KEGG pathway enrichment of shared targets indicated involvement in the cellular senescence pathway (Fig. 6C). Protein–protein interaction (PPI) network constructed via STRING database identified core hub proteins including TP53, AKT1, MAPK1, SIRT1 that functionally converge on cellular senescence regulation (Fig. 6D). Network pharmacology analysis revealed Rhapontigenin (Rhap) as a multi-target agent against senescence and aging.

**Fig. 7:**
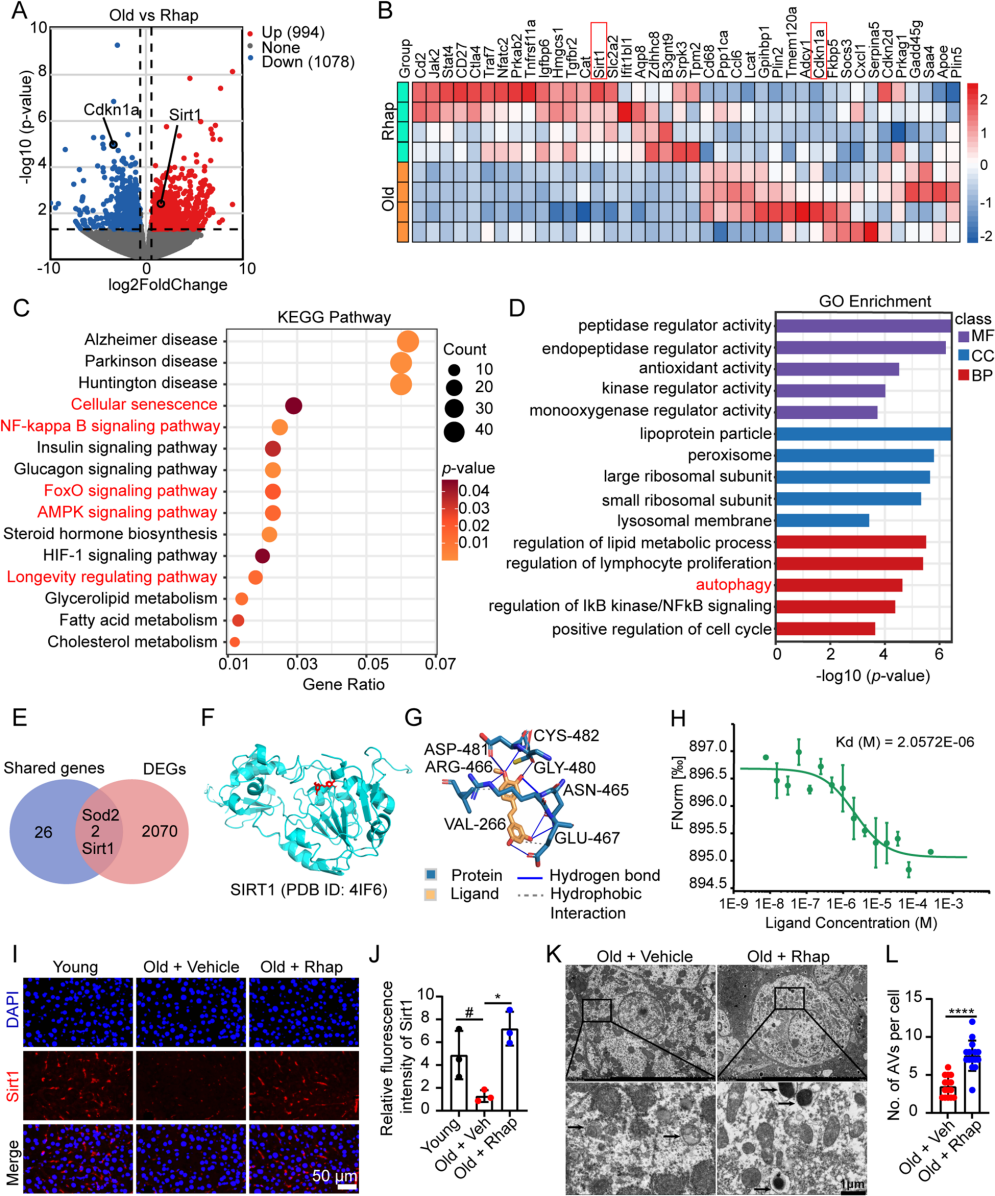
Rhap upregulates Sirt1 expression and promotes autophagy. **A** The volcano plot shows the differentially expressed genes (DEGs) in liver tissues between old mice treated with vehicle and Rhap. **B** Heatmap showing some of the DEGs between old mice treated with vehicle and Rhap. **C** KEGG pathway enrichment of DEGs. **D** GO enrichment in biological process (BP), cellular component (CC), molecular function (MF) of DEGs. **E** Venn Diagram of Overlap between Shared Genes (intersection of Rhap targets, Senescence-related genes, and aging-related Genes) and DEGs. **F** Molecular docking of the Sirt1 protein (PDB ID 4IF6) and Rhap was performed using AutoDock Vina. Docking poses of Rhap (red) in the active site of Sirt1 (blue). The figures were generated by PyMOL. **G** The binding sites of the Sirt1 protein (PDB ID: 4IF6) and Rhap were identified by the PLIP website. The amino acid residues are labeled as indicated. The blue line segments indicate hydrogen bonds. The gray dashed lines indicate hydrophobic interactions. ASP, aspartic acid; ARG, arginine; VAL, valine; GLU, glutamic acid; ASN, asparagine; GLY, glycine; CYS, cystine. **H** The binding capability of Rhap with SIRT1 was examined via MST technology. **I** Representative images of immunofluorescence staining for Sirt1 in liver tissues (scale bar = 50 μm). **J** Quantification of the fluorescence intensity of Sirt1. **K** Autophagosomes and autolysosomes (black arrows) in hepatocytes were observed by transmission electron microscopy (TEM). Representative TEM images of autophagic vacuoles (AVs) are shown. **L** The bar chart shows the number of AVs, including autophagosomes and autolysosomes. The data are presented as the means ± SEM, n = 3–5 mice/group. Statistical analysis was performed with Student’s t test to compare two groups. * *p* < 0.05, *** *p* < 0.001

Transcriptomic analysis of liver tissues revealed significant changes in gene expression following Rhap treatment. Volcano plot analysis identified 2072 differentially expressed genes (DEGs) between vehicle- and Rhap-treated aged mice, with 994 upregulated and 1078 downregulated genes (Fig. 7A). Hierarchical clustering of select DEGs demonstrated clear separation between treatment groups, with distinct expression patterns across biological replicates. Quantitative analysis revealed that Rhap treatment significantly upregulated Sirt1 expression while suppressing transcriptional levels of cell cycle inhibitors Cdkn1a compared with vehicle controls (Fig. 7B). KEGG and GO enrichment analyses of DEGs revealed that Rhap treatment was associated with pathways related to Longevity, Cellular senescence, AMPK signaling, FoxO signaling, NF-κB signaling and autophagy (Fig. 7C, D). Venn analysis identified two critical genes Sirt1 and Sod2 at the shared genes of network pharmacology predictions (Fig. 6B) and DEGs of transcriptomic profiling (Fig. 7E). Considering that Sirt1 is implicated in the regulation of multiple signaling pathways related to aging, such as cellular senescence, longevity regulation, NF-κB, AMPK, FoxOs and Autophagy [35], and exhibits a high degree of connectivity within the PPI network (Fig. 6D), coupled with its upregulated expression in tissues of Rhap-treated aged mice (Fig. 7A, B), we propose the hypothesis that Sirt1 plays a crucial role in mediating the anti-aging effects of Rhap. Molecular docking analysis confirmed that Rhap may interact with the active site of Sirt1 (Fig. 7F, G and Table S3). MST experiments determining the dissociation constant (K_d_) between Rhap and SIRT1 yielded a value of 2.0572E-06 M (Fig. 7H), indicating a strong binding affinity. These findings suggest that Rhap plays a role in modulating Sirt1 activity.

Immunofluorescence staining revealed that Sirt1 expression was significantly greater in the livers of Rhap-treated aged mice than in those of vehicle-treated control mice, suggesting a role of Rhap in promoting Sirt1 activity (Fig. 7I, J). Furthermore, TEM analysis revealed an increase in the number of AVs, including autophagosomes and autolysosomes, in Rhap-treated aged mice, indicating increased autophagy (Fig. 7K, L). These findings suggest that Rhap exerts its antiaging effects, at least in part, by upregulating Sirt1 and promoting autophagy, thereby contributing to improved cellular health in aged mice.

### Rhap reduces cellular senescence by upregulating sirt1 and promoting autophagy

**Fig. 8:**
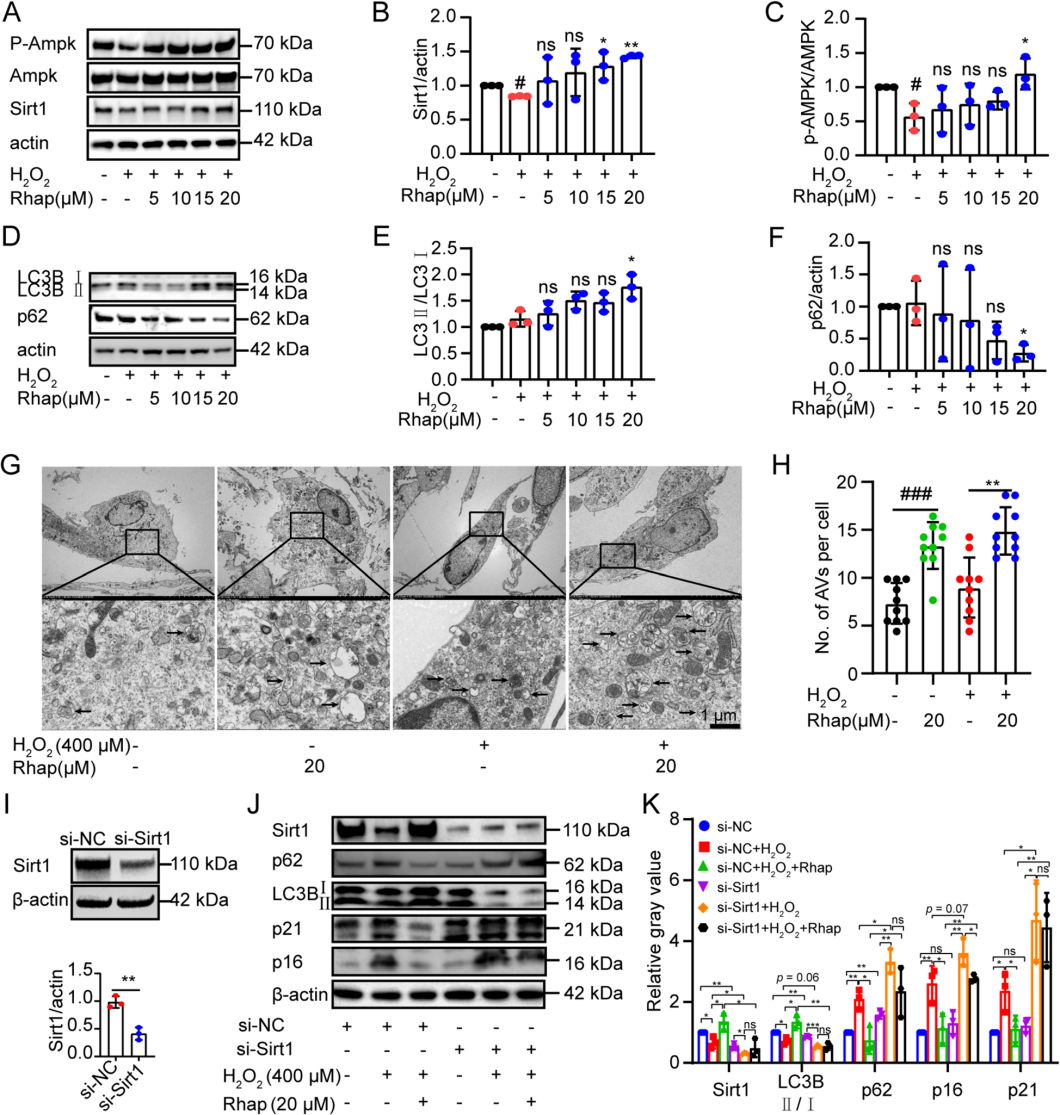
Rhap attenuates cellular senescence in part by upregulating Sirt1 and promoting autophagy. **A**–**C** NIH3T3 cells were cultured without or with 400 μM H_2_O_2_ for 3 h. The next day, the medium was replaced with medium supplemented with 5, 10, 15, or 20 μM Rhap, and the cells were incubated for 48 h. The protein expression of p-Ampk, Ampk, and Sirt1 was measured by Western blotting, and the levels of p-Ampk/Ampk and Sirt1 were quantified. β-actin was used as the reference protein. **D**–**F** The protein expression of LC3B and p62 was measured by Western blotting, and the levels of LC3BII/LC3BI and p62 were quantified. β-actin was used as the reference protein. **G** Autophagosomes and autolysosomes (black arrows) in cells were observed by transmission electron microscopy. **H** The numbers of autophagic vacuoles (AVs), including autophagosomes and autolysosomes, were counted. **I** NIH3T3 cells were transfected with siRNA negative control (si-NC) or an siRNA against sirt1 (si-Sirt1). The protein expression of Sirt1 was measured by Western blotting. The representative results from three repetitions are shown in the figures, and the relative quantities are shown in the scatter bar chart. **J** Western blotting analysis of Sirt1, p62, LC3B, p21, and p16 protein expression in NIH3T3 cells transfected with si-NC or si-Sirt1 followed by treatment with or without H2O2 and Rhap at the concentrations shown in the figure. **K** Bar graph of the relative quantification of the gray values of the protein bands shown in Figure (**J**). The data are presented as the means ± SDs. Statistical analysis was performed with one-way ANOVA with Tukey's multiple comparisons or Student’s t test. * *p* < 0.05, ** *p* < 0.01, *** *p* < 0.001, ns, not significant

To investigate the molecular mechanisms by which Rhap reduces cellular senescence, we examined the role of Sirt1 and autophagy in NIH3T3 cells that were treated with H_2_O_2_ and Rhap. Western blotting analysis revealed that Rhap treatment significantly increased the expression of Sirt1 and the ratio of p-AMPK to AMPK in a dose-dependent manner, indicating AMPK signaling activation (Fig. 8A–C).

Additionally, Rhap treatment increased the LC3BII/LC3BI ratio and decreased the p62 levels, suggesting an increase in the autophagic flux (Fig. 8D–F).

TEM confirmed that the number of AVs, including autophagosomes and autolysosomes, was increased in Rhap-treated cells compared with control cells (Fig. 8G, H). These findings indicate that Rhap promotes autophagy in response to oxidative stress.

To determine whether Sirt1 is required for the anti-senescence effects of Rhap, NIH3T3 cells were transfected with siRNA targeting Sirt1 (si-Sirt1) or a negative control (si-NC). Western blotting analysis revealed that Sirt1 knockdown not only inhibited autophagy and exacerbated H₂O₂-induced senescence in NIH3T3 cells, but also attenuated the Rhap-induced increase in the LC3B-II/LC3B-I ratio and the reduction in p21 and p16 expression. This indicates that Sirt1 is necessary for the Rhap-mediated attenuation of autophagy and senescence (Fig. 8I–K). These findings suggest that Rhap alleviates cellular senescence partly by activating Sirt1 and promoting autophagy, highlighting its therapeutic potential in alleviating age-related cellular dysfunction.

## Discussion

In this study, we demonstrated that Rhap, which is a stilbene aglycone metabolite that is derived from rhaponticin [15], effectively attenuates cellular senescence, increases physical function, reduces inflammation, and promotes autophagy in both in vitro and in vivo models of aging. These findings provide new insights into the potential of Rhap to be used as an antiaging therapeutic agent and contribute to the growing body of literature suggesting a role for natural compounds in mitigating age-associated pathologies.

Rhap is extracted from medicinal plants such as rhubarb rhizomes [36], and exhibit various biological activities, including anti-inflammatory, antioxidant, anticancer, antihyperlipidemic, antiallergic, and antibacterial activities [20, 34, 37–40]. Additionally, Rhap has been reported to scavenge ROS [17] and inhibit DNA, RNA, and protein synthesis in *C. albicans* [16]. Emerging evidence suggests that Rhap exhibits therapeutic potential in mitigating pathological features of Alzheimer's disease (AD) and Parkinson’s Disease (PD), positioning it as a promising therapeutic strategy for AD and PD [41–43]. Our study extends these findings by highlighting the ability of Rhap to counteract oxidative and genotoxic stress-induced cellular senescence, which is a hallmark of aging [44].

The anti-senescence effects of Rhap that were observed in our study are consistent with the findings of previous research on polyphenolic compounds, such as resveratrol and quercetin, which reduce senescence marker levels and improve cellular health by activating Sirt1 and promoting autophagy [45, 46]. Our data show that Rhap significantly reduces the activity of SA-β-Gal and the expression of the key senescence markers p16 and p21 in NIH3T3 and IMR90 cells. Furthermore, direct comparative studies are warranted to rigorously evaluate its potential advantage.

In vivo, Rhap-treated aged mice exhibited significant improvements in physical performance, including enhanced grip strength, improved motor coordination, and reduced depressive-like behaviors. These findings are consistent with those of studies showing that polyphenols can enhance motor function and mitigate mood disturbances during aging [47, 48]. Furthermore, Rhap increased liver health by attenuating hepatic senescence, lipid accumulation, and fibrosis, highlighting its potential for use as an agent for treating age-related liver dysfunction.

Our study also highlights the role of Rhap in modulating immune function. Rhap treatment significantly reduced the levels of proinflammatory cytokines, including IFNγ, TNFα, IL-1α, IL17a and MCP1, which are key components of the SASP. The decrease in the levels of these SASP factors suggests that Rhap can alleviate systemic inflammation and promote a more balanced immune profile.

Mechanistically, our data indicate that the antiaging effects of Rhap are mediated, at least in part, by the upregulation of Sirt1 and the promotion of autophagy. Sirt1 is a well-established regulator of aging, and it is involved in processes such as DNA repair, inflammation, and mitochondrial function [49, 50]. Rhap-treated aged mice exhibited increased Sirt1 expression and autophagy, which support the hypothesis that Rhap promotes cellular homeostasis via autophagy. This finding was further confirmed by our in vitro experiments, in which Sirt1 knockdown not only exacerbated H₂O₂-induced senescence and autophagy dysfunction but, more significantly, markedly attenuated the beneficial effects of Rhap. These results collectively support the model in which Sirt1 acts as a key regulator of the oxidative stress-induced senescence pathway, demonstrating that its genetic depletion aggravates cellular damage. Furthermore, the observed reduction of Rhap’s protective effects upon Sirt1 knockdown suggests that Sirt1 is a critical mediator for the anti-senescence and autophagic functions exerted by Rhap. The dual targeting of the Sirt1 and AMPK signaling pathways by Rhap suggests a multi-faceted mechanism of action, highlighting the potential of Rhap as an antiaging agent.

Despite these promising results, our study has several limitations. We relied primarily on the NIH3T3 and IMR90 cell lines, which may not fully capture the complexity of cellular senescence in vivo. Future studies should include primary cells from aged organisms to confirm the anti-senescence effects of Rhap. Furthermore, our in vitro dose–response analysis could be refined. The fact that Rhap was effective at 5 μmol/L without a stark dose-dependent increase at higher concentrations suggests that the chosen range may have missed the optimal dosing window, and future studies should include lower concentrations to establish a more precise EC₅₀. Additionally, while our study demonstrated the efficacy of Rhap in aged mice, the translation of these findings to humans remains uncertain. Clinical studies are needed to evaluate the safety, bioavailability, and therapeutic potential of Rhap in human populations. Moreover, a detailed pharmacokinetic analysis of Rhap would provide crucial insights into its metabolism and distribution, which are essential for understanding its therapeutic applicability. Finally, while our data strongly suggest that Sirt1 activation is a central mechanism underlying Rhap's effects, the precise molecular pathways by which Rhap upregulates Sirt1 expression and activity remain to be fully elucidated. A more detailed exploration of these mechanisms will be crucial for a deeper understanding of Rhap's action as a natural product.

## Conclusion

In conclusion, Rhap is a promising candidate for mitigating age-related cellular and physiological decline. Our findings suggest that the anti-aging effects of Rhap are largely mediated by the activation of Sirt1 and the subsequent promotion of autophagy, leading to attenuated inflammation and cellular senescence. However, further work is needed to fully elucidate the precise molecular mechanisms of Rhap's action and its pharmacokinetic profile. Future studies are essential to explore its long-term effects, pharmacokinetics, and ultimately, its translational potential in combating age-related pathologies in humans.

## Supplementary Information

Additional file 1.

## Data Availability

The datasets used and/or analysed during the current study are available from the corresponding author on reasonable request.

## Abbreviations

ALT: Alanine aminotransferase
AST: Aspartate transaminase
CHE: Cholinesterase
CREA: Creatinine
IFNγ: Interferon gamma
IL-10: Interleukin-10
IL-1α: Interleukin-1 alpha
IL-1β: Interleukin-1 beta
IL-6: Interleukin-6
MCP-1: Monocyte Chemoattractant Protein-1
Rhap: Rhapontigenin
SA-β-Gal: Senescence-associated β-galactosidase
SASP: Senescence-associated secretory phenotype
Sirt1: Sirtuin 1
TGF-β: Transforming Growth Factor beta
TNFα: Tumor Necrosis Factor alpha
UA: Uric acid
