# Supplementary material for: Rhapontigenin alleviates cellular senescence and physiological aging by upregulating sirt1 and promoting autophagy
Source: Chin Med. 2026 Jan 9;21:22. doi: 10.1186/s13020-025-01319-3 (PMC12784611; doi:10.1186/s13020-025-01319-3)
Supplement: Supplementary file 1 — Additional file 1. [file 13020_2025_1319_MOESM1_ESM.docx]

**Supplementary Materials**

**Rhapontigenin Alleviates Cellular Senescence and Physiological Aging by Upregulating Sirt1 and Promoting Autophagy**

**Shuang Liu^1-3^, Wendi Chen^1-3^, Guoqiang Xu^1-3^, Xin Liu^1-3^, Yuxuan Shi^1-3^, Guolong Wang^1-3^, Yunna Ning^1-3^, Yongzhi Cao^1-3^, Ming Li^4*^, Yueran Zhao^1-4*^**

^1^State Key Laboratory of Reproductive Medicine and Offspring Health, Center for Reproductive Medicine, Institute of Women, Children and Reproductive Health, Shandong University, Jinan, China.

^2^National Research Center for Assisted Reproductive Technology and Reproductive Genetics, Shandong University, Jinan, Shandong, China.

^3^Key Laboratory of Reproductive Endocrinology (Shandong University), Ministry of Education, Jinan, Shandong, China.

^4^Department of Clinical Laboratory, Shandong Provincial Hospital affiliated to Shandong First Medical University, Jinan, China

***Correspondence:**

Yueran Zhao, Email: [yrzhao@sdu.edu.cn](mailto:yrzhao@sdu.edu.cn)

Ming Li, Email: [drmingli@yeah.net](mailto:drmingli@yeah.net)

**Supplementary Materials and Methods**

***Caenorhabditis elegans (C. elegans*) Lifespan Assay**

*Escherichia coli* OP50 cells were seeded on nematode growth medium (NGM) plates and incubated at RT for 24 hours. Synchronized L4-stage N2 worms were transferred to plates containing either the control vehicle (DMSO) or Rhap (0.2 mM or 1.0 mM). The survival of the nematodes was recorded daily using the touch-provoking method. Live worms were transferred to new NGM plates every three days. The experiment was repeated twice, and the data were analyzed using the log rank (Mantel‒Cox) test with GraphPad Prism 8.0.

**Table S1.** Primers used in qPCR.

| Gene | Forward Primer  5’ > 3’ | Reverse Primer  5’ > 3’ |
| --- | --- | --- |
| p16^Ink4a^ | TTCAGGTGATGATGATGGGCAACG | CGGGCGGGAGAAGGTAGTGG |
| p21^Cip1^ | CCGTGGACAGTGAGCAGTTGC | CCCTCCAGCGGCGTCTCC |
| P53 | ACCGCCGACCTATCCTTACCATC | GGCACAAACACGAACCTCAAAGC |
| Il6 | CTTCTTGGGACTGATGCTGGTGAC | AGGTCTGTTGGGAGTGGTATCCTC |
| Il1β | TCGCAGCAGCACATCAACAAGAG | TGCTCATGTCCTCATCCTGGAAGG |
| Mcp1 | TTTTTGTCACCAAGCTCAAGAG | TTCTGATCTCATTTGGTTCCGA |
| Tnfα | ATGTCTCAGCCTCTTCTCATTC | GCTTGTCACTCGAATTTTGAGA |
| Il10 | TTCTTTCAAACAAAGGACCAGC | GCAACCCAAGTAACCCTTAAAG |
| Ifnγ | CTTGAAAGACAATCAGGCCATC | CTTGGCAATACTCATGAATGCA |
| Il1α | GTATGCCTACTCGTCGGGAGGAG | GCAACACGGGCTGGTCTTCTC |
| Tgf-β | CCAGATCCTGTCCAAACTAAGG | CTCTTTAGCATAGTAGTCCGCT |
| Actin-β | CTACCTCATGAAGATCCTGACC | CACAGCTTCTCTTTGATGTCAC |

**Table S2.** Effect of Rhapontigenin on the lifespan of nematode worm.

| Rhapontigenin  (mΜ) | Median life  (days) | Mean lifespan  (days ± SE) | Maximum lifespan  (days) |
| --- | --- | --- | --- |
| 0 | 11.5 | 11.38 ± 0.90 | 20 |
| 0.2 | 14 | 13.85 ± 0.88 | 23 |
| 1.0 | 15 | 13.91 ± 1.07 | 24 |

**Table S3.** Molecule docking scores.

| Receptor name | PDB ID | Ligand name | Affinity(kcal/mol) |
| --- | --- | --- | --- |
| SIRT1 | 4IF6 | Rhapontigenin | -9.9 |
| SIRT1 | 4KXQ | Rhapontigenin | -9.2 |
| SIRT1 | 4I5I | Rhapontigenin | -9.1 |
| SIRT1 | 4ZZJ | Rhapontigenin | -9.0 |

Note: A score of less than -5.0 kcal/mol is considered likely to have docking between ligand and receptor. It is generally accepted that a binding energy <-5.0 kcal/mol indicates good binding activity between the ligand small molecule and the receptor protein, and <-7.0 kcal/mol indicates strong binding activity between the ligand and the receptor.

**
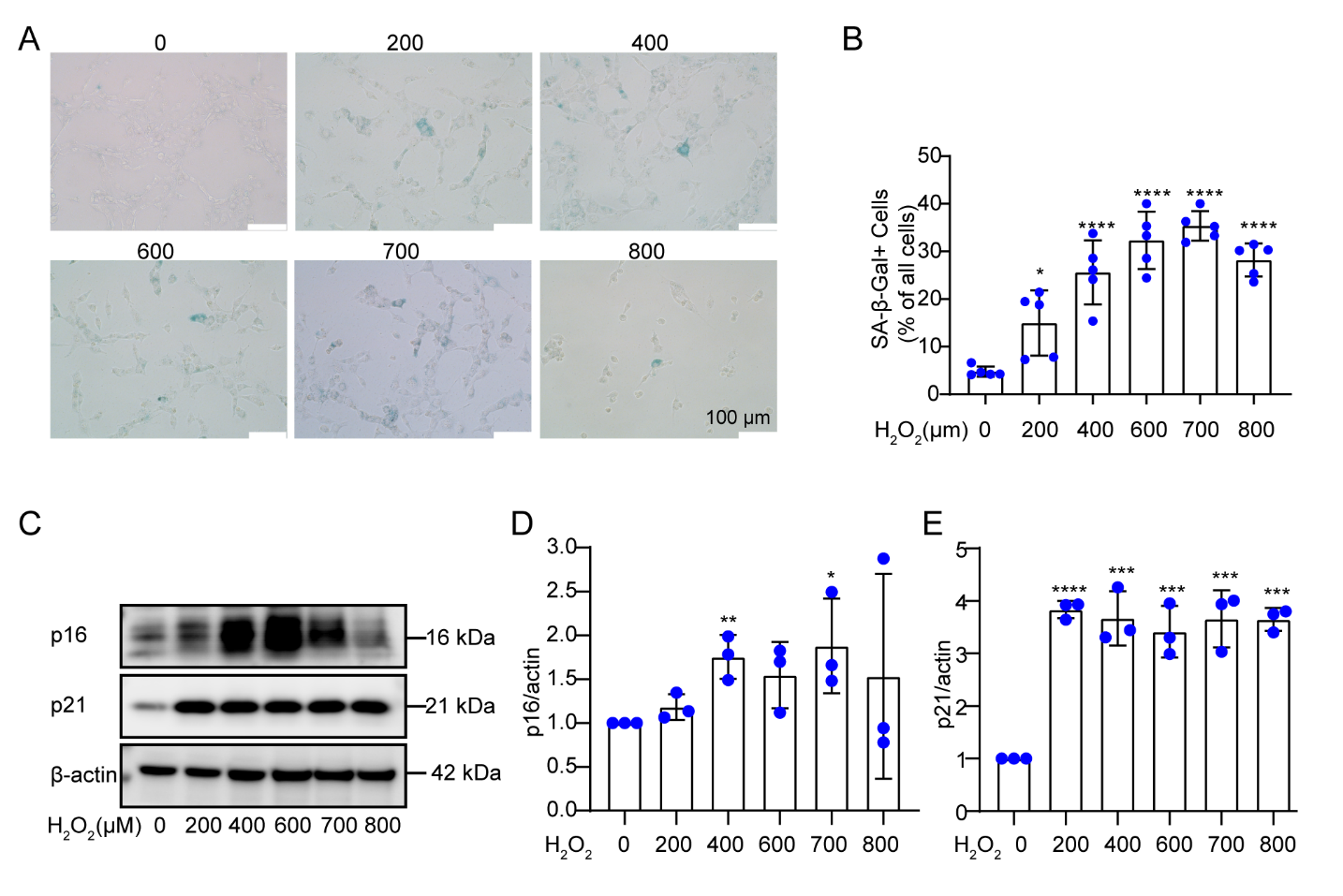
 Figure S1.** Hydrogen peroxide induces senescence in NIH3T3 cells in vitro. Cells were incubated with complete medium supplemented with H_2_O_2_ at the indicated concentrations for 3 h. Then, the medium was replaced with complete medium, and the cells were incubated for 21 h. **(A)** Representative images of SA-β-Gal staining in each group (scale bar = 100 μm). **(B)** Quantification of the percentage of SA-β-Gal-positive cells (5 fields/group, total cells > 250). **(C)** The protein expression of p16 and p21 in H_2_O_2_-treated NIH3T3 cells on day 1 was measured by Western blotting. Beta-actin was used as the loading control. **(D and E)** Quantification of p16 and p21 protein expression in Figure (C). The data are presented as the mean ± SD. Statistical analysis was performed with Student's t test. The group without H_2_O_2_ was used as the control. * *p* < 0.05 vs. the Control group, ** *p* < 0.01 vs. the Control group, *** *p* < 0.001 vs. the Control group, **** *p* < 0.0001 vs. the Control group

**
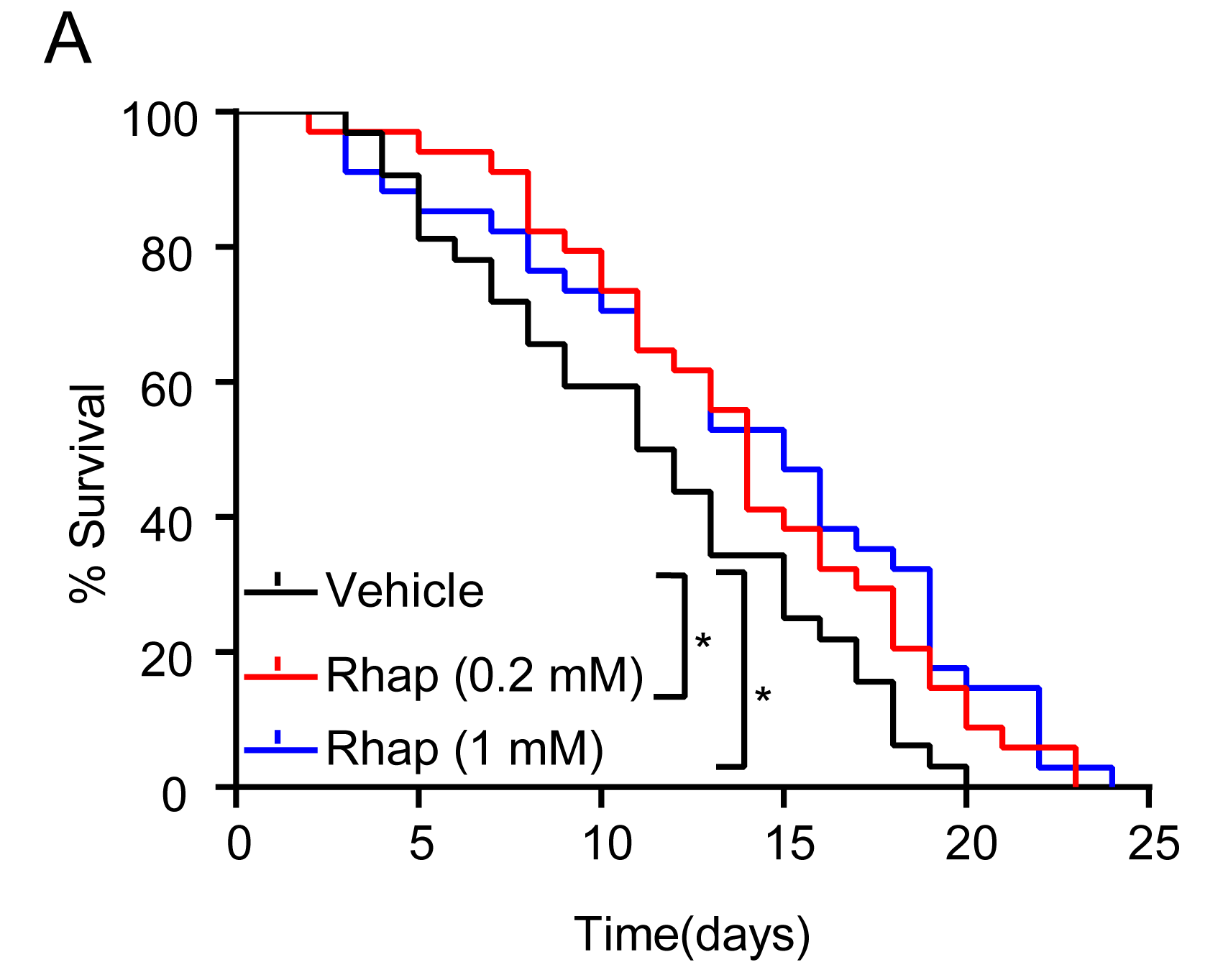
**

**Figure S2.** Rhapontigenin extends the lifespan of N2 nematodes. **(A)** Survival curves of N2 nematodes treated with 0.2 mM or 1 mM Rhap. Statistical analysis was performed using the log rank (Mantel‒Cox) test in GraphPad Prism 8.0. * *p* < 0.05. Rhap, Rhapontigenin

**
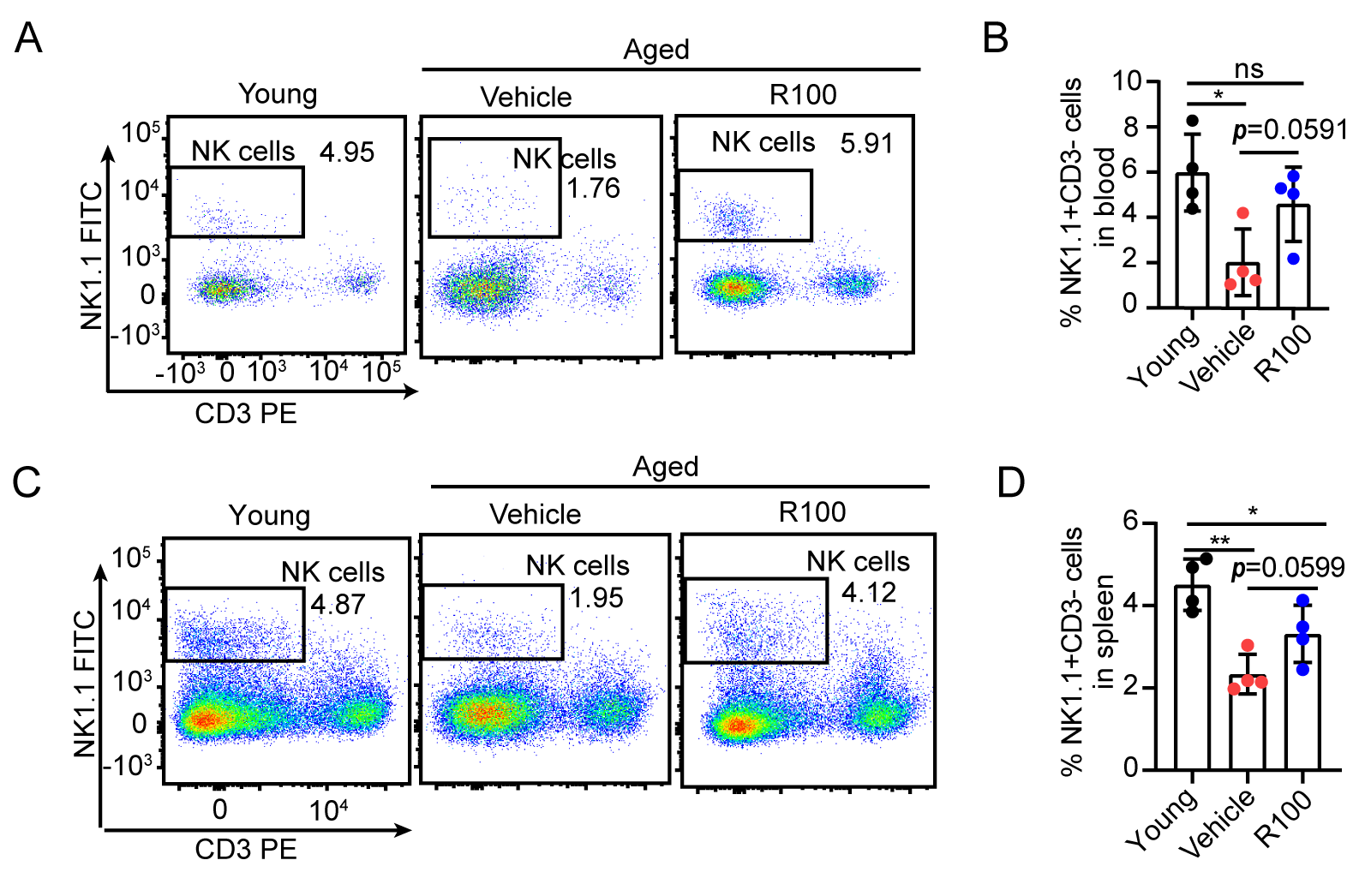
**

**Figure S3.** Rhap have an effect on NK cells. **(A)** Representative flow cytometric plots and **(B)** quantification of the proportion of NK (NK1.1+CD3-) cells in blood samples from the mice. **(C)** Representative flow cytometric plots and **(D)** quantification of the proportion of NK cells in spleen samples from mice. The data are presented as the mean ± SD, n = 4 mice/group. Statistical analysis was performed with one-way ANOVA. * *p* < 0.05, ** *p* < 0.01. R100, Rhapontigenin 100 mg/kg.


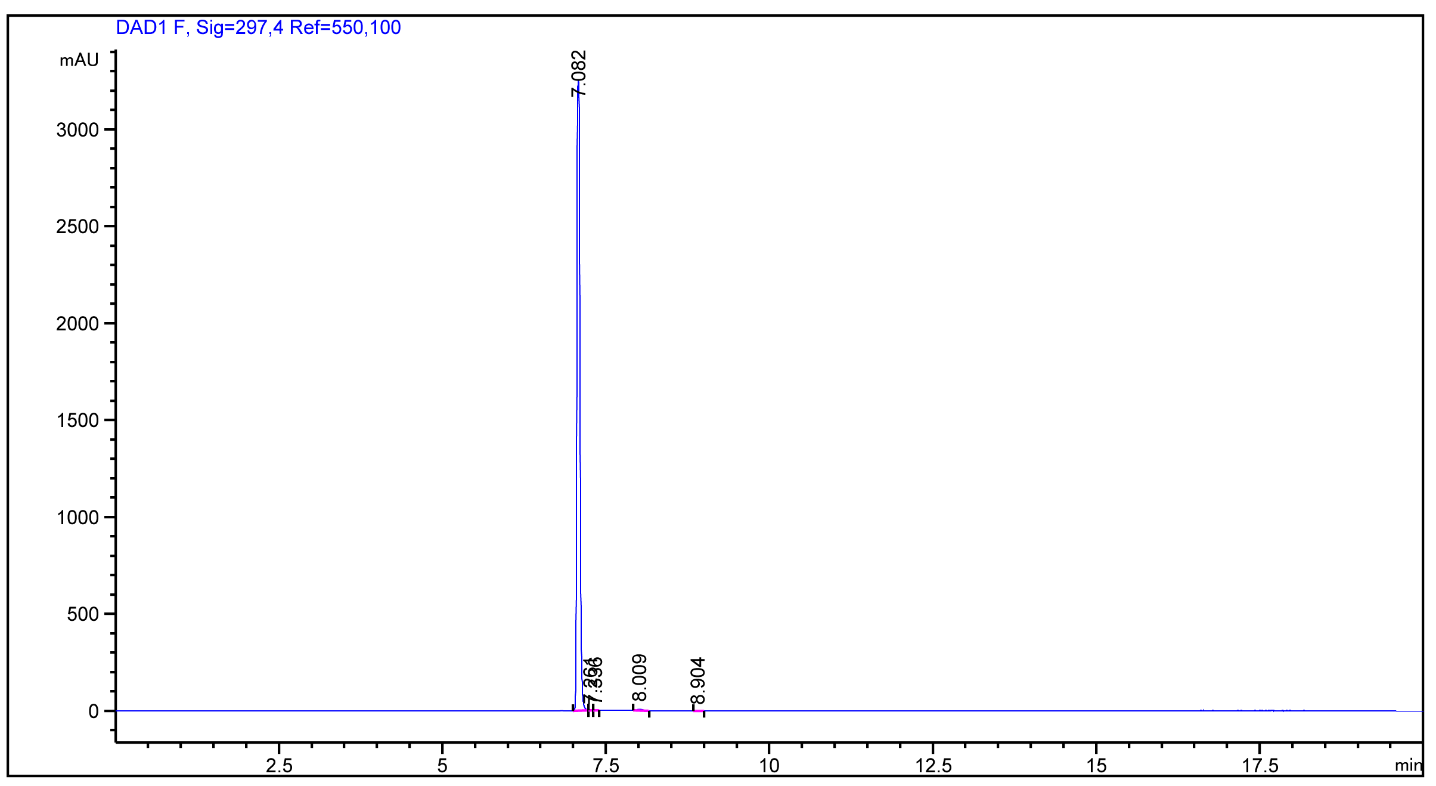


**Figure S4.** HPLC analysis of Rhapontigenin. Purified rhapontigenin was analyzed by reversed phase HPLC on Agilent Zorbax Exlipse Plus C18 column (4.6 mm × 100 mm, 3.5 µm) with a gradient of 10% CH_3_CN (0.1% TFA) to 100% CH_3_CN (0.1% TFA) over 15 min, followed by 100% CH_3_CN (0.1% TFA) for 5 min (flow rate: 1.0 mL/min; detection: UV = 297 nm).
